# Using Wearable Physiological Monitors With Suicidal Adolescent Inpatients: Feasibility and Acceptability Study

**DOI:** 10.2196/13725

**Published:** 2019-09-24

**Authors:** Evan Kleiman, Alexander J Millner, Victoria W Joyce, Carol C Nash, Ralph J Buonopane, Matthew K Nock

**Affiliations:** 1 Rutgers, The State University of New Jersey Department of Psychology Piscataway, NJ United States; 2 Franciscan Hospital for Children Brighton, MA United States; 3 Harvard University Department of Psychology Cambridge, MA United States

**Keywords:** feasibility studies, wearable electronic devices, adolescent, hospitalized, self-injurious behavior, qualitative research

## Abstract

**Background:**

Wearable physiological monitoring devices enable the continuous measurement of human behavior and psychophysiology in the real world. Although such monitors are promising, their availability does not guarantee that participants will continuously wear and interact with them, especially during times of psychological distress.

**Objective:**

This study aimed to evaluate the feasibility and acceptability of using a wearable behavioral and physiological monitor, the Empatica E4, to continuously assess a group of suicidal adolescent inpatients.

**Methods:**

Participants (n=50 adolescent inpatients) were asked to wear an Empatica E4 on their wrist for the duration of their inpatient stay. In addition to assessing behavioral metadata (eg, hours worn per day), we also used qualitative interviews and self-report measures to assess participants’ experience of wearing the monitor.

**Results:**

Results supported the feasibility and acceptability of this approach. Participants wore the monitor for an average of 18 hours a day and reported that despite sometimes finding the monitor uncomfortable, they did not mind wearing it. Many of the participants noted that the part of the study they enjoyed most was contributing to scientific understanding, especially if it could help people similar to them in the future.

**Conclusions:**

These findings provide promising support for using wearable monitors in clinical samples in future studies, especially if participants are invested in being part of a research study.

## Introduction

Objective measures of physiological factors such as electrodermal activity (EDA) and heart rate (HR) have existed for over 100 years. Shortly after their development, researchers started using these tools in laboratory settings to examine the association between physiology and emotion (for historical reviews, see studies by AlGhatrif et al [1], Fye [2], and Neumann and Blanton [3]). Recent technological advances have enabled the study of human behavior and psychophysiology outside the laboratory, in the real world, using research-grade wrist-worn physiological monitors. These monitors enable the continuous, extended, real-world assessment of many of the same constructs once only possible to assess in laboratory settings over short assessment periods [4,5]. This offers great promise to improve our understanding, prediction, and prevention of factors related to psychological phenomena of interest. One area where technology may be particularly useful is suicidal thoughts and behaviors, which are highly prevalent among adolescents (15% of all adolescents each year seriously consider suicide [6]) and present increased risk for suicide death, which account for 8.5% of all deaths among adolescents and young adults globally [7]. For example, through other technology-based studies (ie, studies using mobile phone app-based ecological momentary assessment), we know that suicidal thinking varies rapidly throughout the day and is associated with times of intense psychological distress (ie, high-arousal negative affect) [8]. We also know from laboratory studies that physiological signals such as EDA and HR map on to psychological distress [9-13]. We do not know, however, whether these EDA and HR are associated with suicidal thinking as it occurs in real time. Having this information would allow us to create interventions that can be trigged based on these physiological signals and delivered just in time as needed.

Public familiarity with wearables has increased in recent years with availability of consumer-grade monitors (eg, devices made by Fitbit and Apple). Unfortunately, these commercial monitors are limited for research use, given that they far are less accurate than gold-standard laboratory-based monitors [14-16] and often do not include sensors to measure important psychophysiological variables such as EDA. Compared with consumer-grade devices, scientific-grade wearable monitors are more accurate but also more expensive, bulkier, and less user-friendly than consumer-grade wearables. Thus, we cannot infer that high acceptability of consumer-grade wearables will translate to research-grade monitors. Accordingly, before such widescale research is possible, it is important to assess the feasibility and acceptability of using these monitors in real-world research. The goal of this study was to assess feasibility and acceptability of a commonly used research-grade wearable physiological monitor (Empatica E4, Empatica Srl) to continually assess behavior and psychophysiology among a clinically severe group of adolescents—those hospitalized for suicidal thoughts and behaviors—over the course of their hospital stay. The Empatica E4 is a physiological monitor that is worn on the wrist like a watch and records several streams of physiological data, including EDA and HR (through an optical sensor), as well as temperature and movement (through an accelerometer).

There are 3 important reasons to evaluate the feasibility and acceptability of these monitors within adolescent and psychiatric populations. First, previous studies examining the feasibility of wearable physiological monitors such as the E4 have been among adult samples that are relatively psychologically healthy, such as people who suffer from migraines [17] and tourists visiting a new city [18]. These studies cannot tell us whether adolescents (who may find new technology more acceptable or may be more self-conscious about the aesthetics of a wearable monitor) and those with more severe psychopathology (whose psychopathology may create competing demands for cognitive resources) would find these monitors acceptable. Second, nearly all studies have only collected data for a short period of time (eg, 10-20 min [19,20]) and cannot tell us whether participants would find it acceptable to use the monitor over far longer periods (eg, days, weeks, or months). Third, some newer wearable monitors are equipped with an event marker button that participants can use to report the experience of some psychological events/outcomes of interest. Such a feature allows researchers to examine physiological data leading up to (and following) events of interest. However, there has been no exploration of whether it is feasible to ask adolescent participants to press an event marker button during times of transient psychological events (eg, intense distress and severe suicidal thinking).

To our knowledge, only 1 study directly addressed feasibility/acceptability of the E4 [21]. This study found that a group of adults with schizophrenia and a control group were able to follow the instructions for using the E4 and rated it highly on a composite measure of acceptability. Although this information is useful, it leaves unaddressed several questions about the feasibility and acceptability of using such monitors with adolescents in acute psychiatric distress and over longer periods. Thus, here, we were interested in 3 questions relating to feasibility and acceptability that have not been addressed by previous studies.

First, we wanted to examine whether participants would wear the monitor the majority of each day over the course of multiple days. A monitor that can assess psychophysiology continuously throughout the day is only useful if participants are willing to wear the monitor over this period. Previous work has generally lasted only a few hours and therefore could not examine whether participants are willing to continuously wear the monitor over days or weeks. Second, we were interested in whether participants would interact with the monitor (ie, use the self-initiated button press). Simply collecting behavioral and psychophysiological data does not enable researchers to document (and predict) behavioral or cognitive outcomes (eg, psychological distress, suicidal thoughts, and hallucinations). However, such a button is only useful if participants remember to press the button when they should and do not press the button when they should not. One previous study presents some data on whether adolescent participants could reliably press an event marker on a wearable monitor (actigraphy watch [22]) each time they laid down to rest/sleep or got up from resting/sleeping. Although the authors of this study did not report actual compliance rates regarding the use of the event marker, they noted that “the event marker button was reliably used during the first few days but, afterward, some participants neglected to use it.” The experience of transient psychological events such as intense distress may be more (or less) memorable than times before and after rest, and thus, it is unknown whether participants will use the event marker at the same rate as this previous study. Third, we were interested in what participants liked (or disliked) about wearing the monitor. The only study to explicitly address participants’ opinions on the monitor [21] reported only that approximately 80% of their entire sample rated the monitor as *good* or *excellent* but did not report what participants specifically liked or disliked about the monitor.

## Methods

### Participants

Data were drawn from the first 50 participants from a larger, ongoing study of suicidal adolescent inpatients assessing the risk of harm to self or others using wearable ambulatory monitoring. Participants were recruited from a large urban inpatient psychiatry unit. Inclusion criteria for the study were (1) admitted for risk of harm to self (eg, severe suicidal ideation, suicide attempt, and nonsuicidal self-injury), (2) being aged 12 to 19 years, and (3) having at least one wrist with unbroken skin where the wristband could be placed.

### Recruitment and Data Collection Procedures

The study took place during participants’ inpatient stay. Owing to hospital policy, we were not able to compensate participants.

#### Consent

We recruited and consented participants as close to hospital admission as possible. For potential participants aged younger than 18 years, we first approached parents/guardians to get their written consent and then approached the participant to get their written assent. We directly approached potential participants who were aged 18 years or older and received written consent. All study procedures were approved by the governing hospital and university institutional review boards.

#### Baseline Measures and Wearable Monitor Training

After providing informed consent, participants completed a brief set of self-report measures as part of the larger study but not relevant to this study and so not discussed further here (eg, measures of emotion regulation and impulsiveness). Next, participants completed a brief training session on how to properly wear and use the E4 and received a laminated 1-page information sheet with the same instructions to serve as a personal reference.

#### Monitoring Period

For the duration of the inpatient stay, we asked participants to wear an E4 on their dominant wrist as often as possible (eg, during the day and while sleeping) as long as the monitor was not at risk of getting wet (eg, during showers). The E4 has an event marker button that can be used to *tag* events defined by the research team. We asked participants to press the marker button on the E4 whenever they felt distressed, which we defined as “Feeling so upset or angry that you have an urge to hurt yourself or someone else or to break something.” We made sure that participants were aware that no one was actively monitoring when they pressed the button (ie, pressing the button would not signal the clinical team to come help them, and we would not share the study information with the clinical team). Each day, a study staff member (during the workweek) or a clinical staff member (during the weekend) switched each participant’s E4 for a fully charged monitor.

#### Daily Check-In Surveys

Each weekday, a study staff member approached the participant to conduct a brief check-in about any problems with the E4 that occurred since the last check-in. Staff members also assessed (1) whether the participant recalled missing any occasions when they believe they should have pressed the button but did not and (2) whether the participant accidentally pressed the button. If a participant recalled missing a button press or accidentally pressed the button, we assessed when and why this occurred.

#### Discharge

Shortly before their discharge from the hospital, participants completed 2 sets of open-ended questions aimed at assessing their experiences wearing the E4. First, they completed a 12-item questionnaire regarding satisfaction with the E4, modeled after other measures of comfort with wearable devices, specifically the Wearable Computer Comfort Rating Scale developed by Knight and Baber [23]. Items on this measure assessed (1) concerns about appearance when wearing the device (eg, “I felt anxious wearing the device”), (2) the physical feel of the device (eg, “The device was uncomfortable to wear”), (3) whether the device affected movement (eg, “the device made it hard to sleep at night”), and (4) general worries about taking care of the device (eg, “I worried about taking care of the device”). All items were on a 0 (never) to 10 (all the time) scale. Second, participants completed 4 open-ended qualitative questions: (1) “What did you like MOST about wearing the device?” (2) “What did you like LEAST about wearing the device?” (3) “How did you feel when wearing the device?” and (4) “Is there anything you would change about the device?” Due to reasons unrelated from the study (eg, discharge came quicker than expected), 3 out of the 50 participants were not able to complete the qualitative assessment.

### The Wearable Monitor

#### Overview

The Empatica E4 (Empatica Srl) is a research-grade wrist-worn behavioral and psychophysiological monitor. Its case is 44 mm long (~1.73 inches), 40 mm wide, and 16 mm deep. This means that it is larger than commercially available wearable monitors (eg, the Fitbit Charge HR 2 is 22.86 mm long, 12.7 mm wide, and 11.0 mm deep). It has 4 main sensors: (1) a light-emitting diode–based photoplethysmograph (PPG) used to derive HR from blood flow, (2) a pair of silver-plated EDA/skin conductance sensors, (3) a 3-axis accelerometer, and (4) an infrared thermopile used to determine temperature. The E4 collects these data in real time and stores them on the onboard flash memory (which can hold ~60 hours of data, at 1 MB per hour). The E4 is then connected to a computer through a universal serial bus cradle and synchronized to a secure cloud server through the *E4 Connect* software. The E4 has a 250 mAh battery (lasting ~36 hours) that charges through the synchronizing cradle. The E4 also offers Bluetooth streaming, which can transmit data to the cloud using a compatible mobile phone as a gateway. We did not use this option because it would not have been feasible for long-term use owing to increased battery consumption as a result of using the Bluetooth radio and limitations in mobility (participants would need to be <30 feet from a mobile phone at any time).

#### Placement of E4 on Dominant Wrist

The larger goal of this study was to test whether the physiological and behavioral data collected from the E4 can accurately predict episodes of self-directed and other-directed violence. Thus, we made the decision about which wrist to wear the E4 based on our goal of collecting this data stream. There is currently a debate in the field about which wrist is optimal for assessing EDA. A body of work suggests that high arousal negative emotion (eg, distress) can be detected through examining asymmetry between the left and right sides of the body [24], with more pronounced signals coming from the dominant side. Given that wearing 2 monitors at once would be cumbersome for participants, we elected to have participants wear the monitor on their dominant wrist, which would likely provide the most pronounced changes in EDA in response to distress. This is also in line with the manufacturer’s recommendation [25]. It is important to note, however, that the dominant wrist likely produces noisier data (because of motion artifacts) than the nondominant wrist. Thus, researchers should examine the trade-off between signal and noise when choosing which wrist to use in their own work.

#### Logistics of Charging and Synchronizing E4

Each participant was assigned 2 wristbands but only wore 1 wristband at a time. While the participant wore 1 wristband, the other was placed on a dock to charge and synchronize data. We assigned participants 2 wristbands because charging and synchronizing the E4 can, in some cases, take more than an hour. Thus, having just 1 E4 per participant would have meant that we would lose more than an hour of data per day while the monitor was charging. As the E4 software can only synchronize 2 E4s at a time, it would have been cumbersome to have only 1 monitor per participant because it would have required study staff to rotate the E4 on the synchronizing cradle throughout the day. To accommodate a larger number of participants (we had up to 10 simultaneously), we constructed a charging and synchronizing station that we could place the monitors on when they were not being used, which was kept in a research office adjacent to the inpatient unit. The monitors would charge simultaneously and synchronize consecutively (ie, when 1 E4 was finished synchronizing, the computer moved on to the next one).

### Analytic Strategy

#### How Often Will Participants Wear the Monitors?

To answer this question, we calculated 2 features from the E4’s recording length metadata: (1) number of days that each participant wore the monitor for at least some amount of time and (2) number of hours during each day that participants wore the monitor. From these features, we calculated the intraclass correlation (ICC) of hours per day wearing the monitor. This allowed us to examine how much of the variability in time wearing the band was because of between-person differences (ie, whether some participants consistently tended to wear the band for more or less time than other participants) versus within-person differences (ie, whether participants wore the band a lot more or less frequently on some days than other days). We also compared these features by attrition status (ie, between those who did and did not drop out of the study).

#### Do Participants Correctly Use the Event Marker?

To answer this question, we calculated 3 sets of features: (1) number of button presses extracted from the raw data from the tags.csv file on the E4, (2) the number of missed button presses (and the reasons for missed press) extracted from the daily participant surveys, and (3) the number of accidental button presses (and reasons for the accidental press) extracted from the daily surveys. We also examined the ICC for the number of button presses each day, again to determine whether some patients tended to press the button more often than others or if patients pressed the buttons more on some days than others.

#### Do Participants Like Wearing the Monitor?

To answer this question with the quantitative data, we calculated the means and SDs for each item on the comfort assessment measure. We explored the association between each item on the measure and length of time wearing the band each day. We also compared responses on the measure between those who did and did not drop out of the study. Both sets of comparisons were corrected for multiple comparisons using a Bonferroni correction. We used a 3-step process to answer this question using qualitative data. First, the first author did a line-by-line read-through of the responses to the 4 qualitative questions and developed a codebook (described below). Second, the first, second, and last authors independently coded the responses based on this codebook. Third, we resolved any discrepancies to reach a final consensus. As with the quantitative data, we also examined associations between the qualitative data (ie, differences by whether participants did or did not endorse a qualitative category) and length of time wearing the monitor each day (using a *t* test) and in dropout status (using a chi-square test).

## Results

### Descriptive Statistics

Participants’ age ranged from 12.5 to 18.6 years (mean 16.3, SD 1.6), 78% (39/50) of the sample was female and 92% (46/50) of the sample was white (4%, 2/50 was Asian and the remainder indicated that they identified with another race). Participants together provided 487 total days of data (mean 9.74 days per participant, SD 13.81 days, range 1-76 days). The average length of stay in the hospital (ie, day of intake to day of discharge) was 10.7 days (SD 13.86 days, range 1-77 days). There were no demographic differences by age or sex (there were too few cases to examine racial differences) on any key study variables including hours wearing the monitor per day (age: *r*=0.05, *P*=.77 sex: *t*_11.12_ *(two-tailed)*=0.13, *P*=.90), number of button presses (age: *r*=0.10, *P*=.58; sex: *t*_23.94_ *(two-tailed)*=1.26, *P*=.22), and dropout status (age: *t*_5.81_ *(two-tailed)*=0.09, *P*=.93; sex: χ^2^_1_=0.8, *P*=.38).

### Do Participants Wear the Monitor?

**Figure 1:**
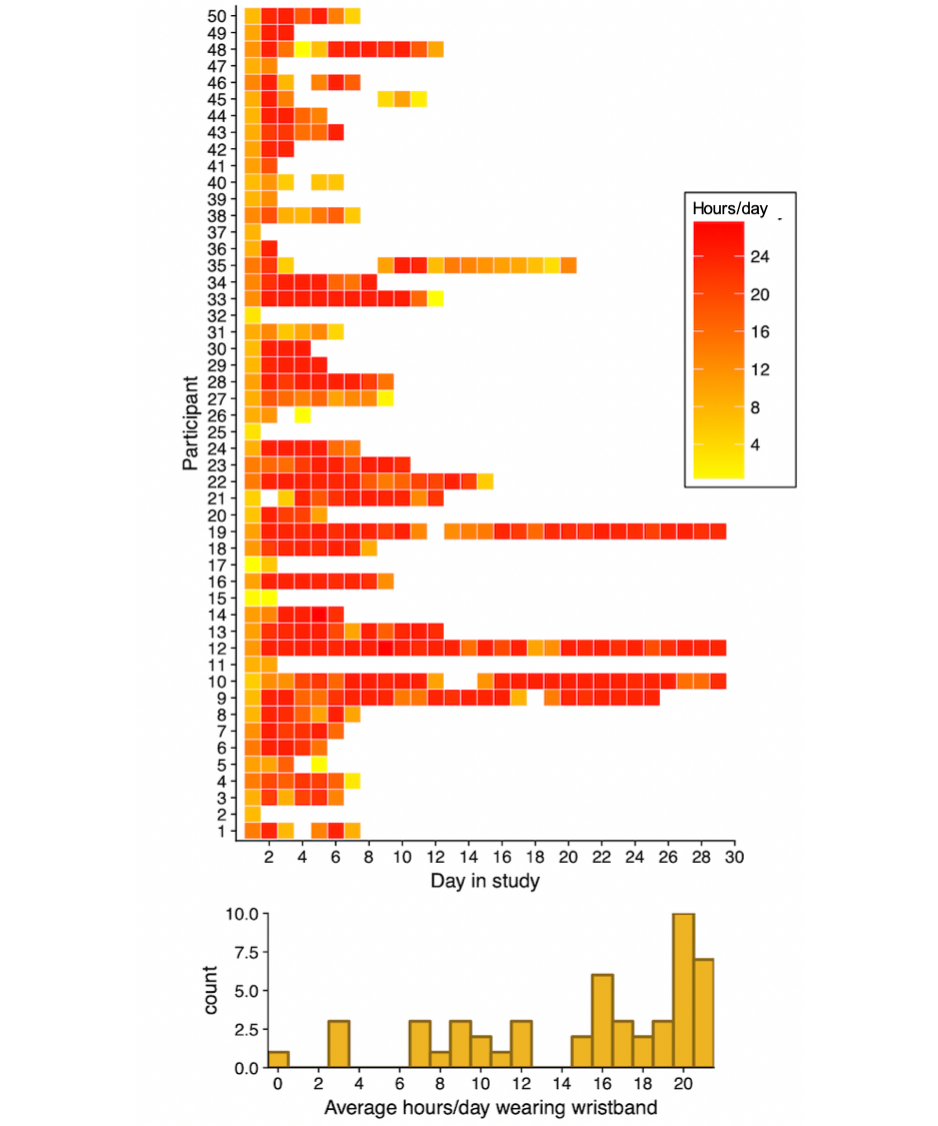
Total daily hours worn each day (top) and histogram of average time worn each day per participant (bottom). White squares in top panel: band not worn that day. Participants marked in gray: dropouts. For clarity, range truncated to 1 to 30 in top panel (7 values [1.5% of all responses >30]). Count in bottom panel refers to number of participants.

Participants wore the E4 at some point during the day (ie, any nonzero amount of data for the day) for 464 of a possible 487 total study days (95.3% of all days; see Figure 1, top panel). The majority (15/23; 65%) of days with no data were from 1 participant (#10 in the figures), although this participant was in the study for the longest amount of time (76 days). As can be seen in the bottom panel of Figure 1, there was considerable variability in the average number of hours each participant wore the band each day. On days when participants wore the E4, they did so for an average of 18.3 hours (SD 6.3). Excluding the first and last days of the study, when participants would not have been able to wear the monitor for 24 hours, participants wore the monitor for an average of 20.3 hours (SD 5.3). There was no association between day in study and hours worn (slope [*b*]=−0.10; *P*=.46), suggesting that participants did not wear the monitor any more or any less as their time in the study increased. When visually inspecting the scatterplot of this association, there was no clear nonlinear effect, suggesting that participants did not likely wear the band more toward the middle of the study than at the end. Regarding variability in hours worn per day, there was more within-person (ie, day-to-day) variability in hours worn per day than there was between-person variability (ICC=0.31, 95% CI 0.21-0.43). This means that one-third of the variability was because of some participants tending to wear the E4 longer than others, whereas two-thirds of the variation in the amount of time wearing the monitor was accounted for by day-to-day variation within people.

#### Dropout

Overall, 7 participants discontinued wearing the monitor before they were discharged. As expected, those who dropped out of the study participated for significantly fewer days (mean 2.1, SD 1.4) than those who continued the study until they were about to be discharged from the hospital (mean 11.2, SD 14.7; *t*_45.66_=3.92, *P*<.001) and for significantly fewer hours each day when they were in the study (mean 7.4, SD 6.3) than those who continued in the study (mean 18.7, SD 6.5; *t*_13.88_=6.61, *P*<.001).

### Do Participants Correctly Use the Event Marker?

**Figure 2:**
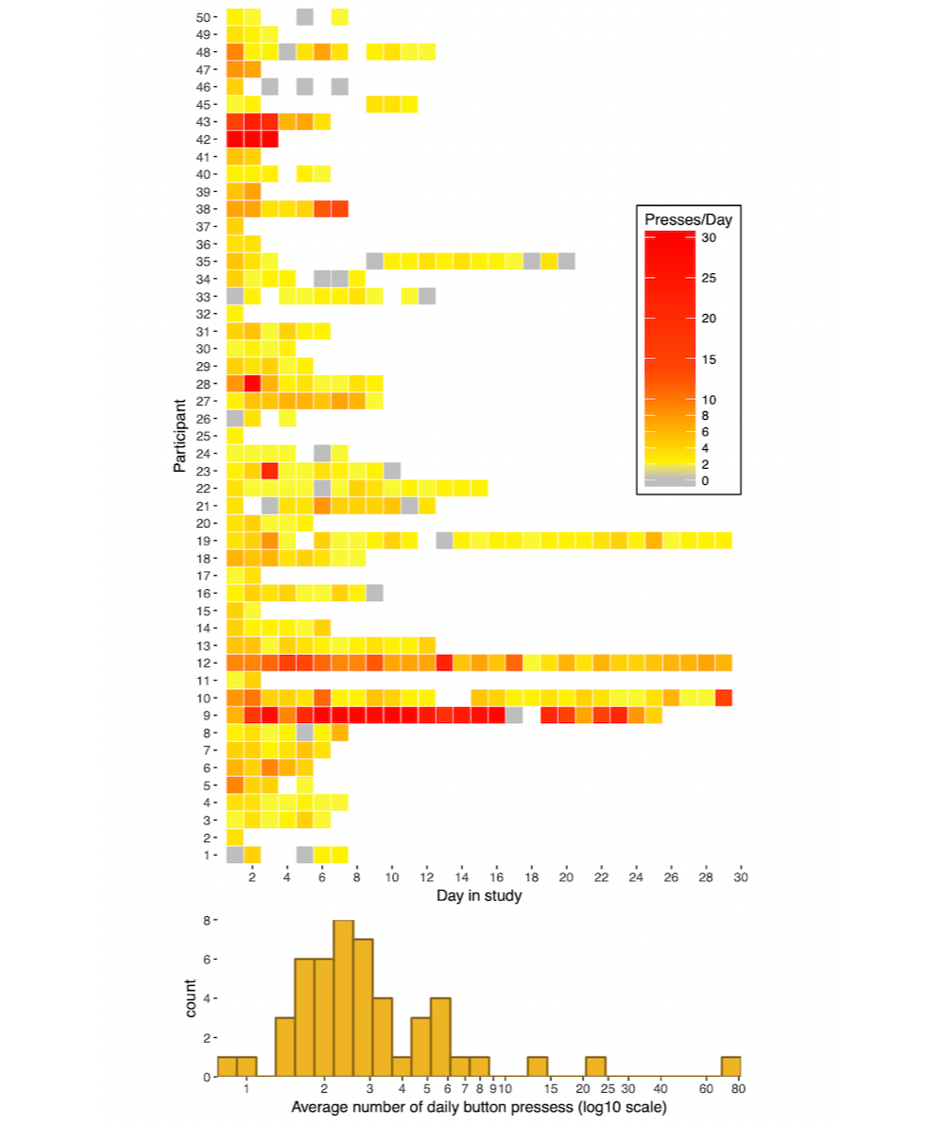
Total daily button presses (top) and histogram of average button presses per participant (bottom). White squares in top panel: band not worn that day. Participants marked in gray: dropouts. For clarity, range truncated to 1 to 30 days in top panel (7 values [1.5% of all responses >30]). Count in bottom panel refers to number of participants.

There were 2159 button presses (ie, uses of the event marker) recorded during the study, which occurred during 435 of the 464 days (93.8%) during which participants wore the monitor. Participants pressed the button on average 4.9 times per day (SD 9.3, range 0-140). The top panel of Figure 2 shows a plot of the daily number of button presses for each participant during each day they were in the study.

As can be seen in the bottom panel of Figure 2, there was considerable variability in the average number of presses per participant per day ranging from 0.8 presses per day to 77.7 presses per day. In contrast to the amount of time wearing the monitor, there was more between-person variability in average number of button presses per day than there was within-person variability (ICC=0.63, 95% CI 0.52-0.73). This means that most of the variability in button presses was between-individuals, and each participant tended to stay near their own average throughout the study. As would be expected, when looking within each day regardless of subject, there was a small but significant association between hours worn per day and number of button presses (*b*=0.09, 95% CI 0.01-0.16; *P*=.04). When examining the association between day in study and number of times pressing the button, there was a small but statistically significant association *(b*=−0.10, 95% CI −0.15 to −0.05; *P*<.001), suggesting that as participants were in the study for a longer period of time, they pressed the button slightly less.

#### Missed Presses

There were 40 total missed button presses reported during the study. Of 40 missed presses, 17 came from 1 participant. The remaining 23 missed presses (57% of all presses) came from 14 participants (mean 1.7 presses per participant, SD 1.3, range 1-6). Regarding reasons for missed presses, nearly all the missed presses (32/40; 80% of presses) were because of the participant forgetting to press the button. The remainder of missed presses were because they were not wearing the monitor or the battery had died (4/40; 10% of presses) or because of an inability to pick a specific point when feeling distressed (2/40; 5% of presses) or misunderstanding that they were supposed to press the button (2/40; 5% of presses).

#### Accidental Presses

There were 10 accidental button presses reported during the study (0.46% of all presses). No participant had more than 1 accidental button press. The reasons participants accidentally pressed the button fell into 2 broad categories: (1) another patient pressed their button (6/10 presses) and (2) the result of trying to turn off the monitor by holding down the button, but accidentally releasing it too early (3/10). For the 1 remaining accidental press, the patient did not recall the circumstances.

### Do Participants Like Wearing the Monitor?

#### Quantitative Measures

**Figure 3:**
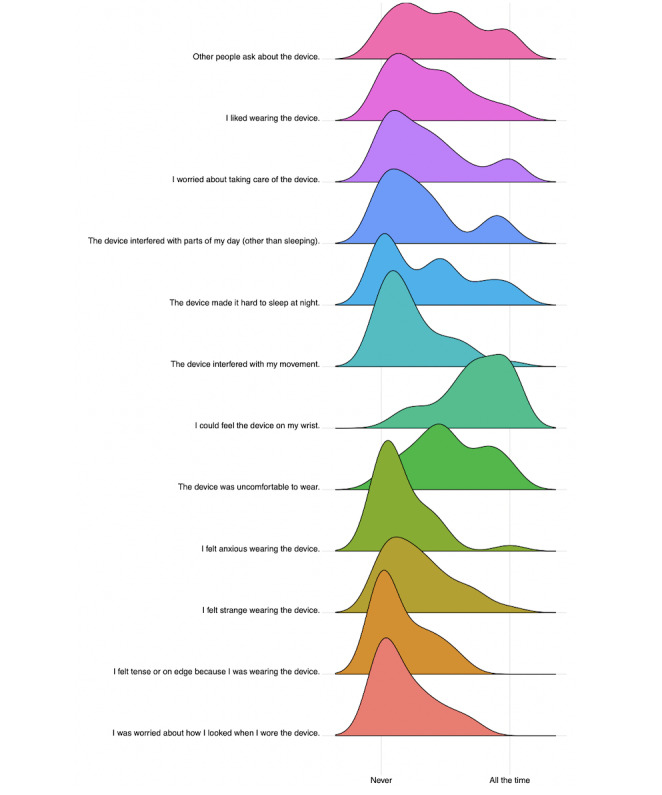
Distribution of quantitative response to wearable comfort measure.

A summary of responses to the quantitative questions about device comfort is presented in Table 1, and a visualization of the distribution of responses is shown in Figure 3. On average, across nearly all items assessing comfort, participants tended to rate their discomfort wearing the monitor below 5 out of 10 (with 10 meaning more discomfort). As can be seen in the middle columns of Table 1, there was a significant negative correlation between hours worn and ratings of how uncomfortable the monitor was, such that the more participants rated the monitor as uncomfortable, the fewer hours they wore it. No other correlations were significant after correcting for multiple comparisons. As can be seen in the rightmost columns of Table 1, after correcting for multiple comparisons, there were only 2 significant findings: (1) those who dropped out were significantly more likely to rate the device as uncomfortable and (2) were more likely to note that they could feel the device. The qualitative data illustrated these quantitative findings well. One participant who dropped out said, “I really wanted to keep it on so I could help but it was too uncomfortable,” and another said, “I could feel the silver [EDA electrodes] rubbing.”

**Table 1:** Quantitative assessment of wearable monitor comfort.

Item	Descriptive, mean (SD)	Correlation with time worn		Attrition status			
		*R* value	*P* value	Did not drop out, mean (SD)	Dropped out, mean (SD)	Comparison	
						*t* test *(df)*	*P* value
							
I was worried about how I looked when I wore the device.	1.87 (2.26)	−0.14	.45	1.93 (2.29)	1.33 (2.31)	0.42 *(2.44)*	.71
I felt tense or on edge because I was wearing the device.	1.59 (2.05)	−0.04	.82	1.66 (2.13)	1.00 (1.00)	0.94 *(4.24)*	.40
I felt strange wearing the device.	2.91 (2.67)	−0.19	.26	2.65 (2.35)	5.00 (4.40)	−1.05 *(3.22)*	.36
I felt anxious wearing the device.	1.63 (2.22)	0.07	.73	1.78 (2.29)	0.33 (0.58)	2.61 *(12.25)*	.02
The device was uncomfortable to wear.	5.23 (2.90)	−0.49^a^	.001^a^	4.53 (2.60)^a^	8.86 (1.07)^a^	−7.30^a^ *(22.64)*	.001^a^
I could feel the device on my wrist.	7.28 (2.62)	−0.26	.08	6.87 (2.63)^a^	9.57 (0.79)^a^	−5.24^a^ *(33.11)*	.001^a^
The device interfered with my movement.	2.35 (2.55)	−0.13	.47	2.04 (2.13)	3.83 (3.87)	−1.10.*(5.67)*	.31
The device made it hard to sleep at night.	3.53 (3.50)	−0.07	.68	3.50 (3.38)	3.67 (4.46)	−0.09.*(6.12)*	.93
The device interfered with parts of my day.	3.15 (3.20)	−0.31	.08	2.67 (2.95)	5.33 (3.67)	−1.66.*(6.51)*	.14
I worried about taking care of the device.	3.51 (3.28)	0.02	.90	3.59 (3.20)	3.00 (4.12)	0.31 *(4.73)*	.77
I liked wearing the device.	3.60 (2.96)	0.30	.05	3.66 (2.97)	3.29 (3.15)	0.29 *(8.10)*	.78
Other people ask about the device.	4.46 (3.27)	0.33	.04	4.69 (3.14)	2.80 (4.09)	1.00 *(4.70)*	.37

^a^Values are significant after Bonferroni correction (0.05/24=0.002).

#### Qualitative Measures

The initial read-through of the qualitative responses yielded 13 codes across 3 categories: complaints about the monitor (7 codes), positive/neutral statements about the monitor (3 codes), and positive states about the study itself (3 codes). The results of the qualitative analyses are shown in the middle columns of Table 2. Our initial reliability across all codes was acceptable (kappa=0.77, SD 0.20, range 0.041-1.00). The 3 raters were able to come to a consensus on all the discrepancies. The rightmost column of Table 2 shows the frequency with which each code was endorsed and example statements from each code.

**Table 2:** Results of qualitative analyses (N=47).

Category		Example	κ^a^ value	*Z* value	Endorsed, n (%)
**Complaints about monitor**					
	Discomfort/uncomfortable in general	“It felt extremely uncomfortable.”	0.68	8.08	22 (47)
	The monitor was too big/clunky	“It was bulky and inconvenient.”	0.9	10.74	17 (36)
	It should have a clock	“I would probably make it have a clock.”	0.92	10.89	11 (23)
	The material was uncomfortable	“I’d make the wristband out of a thinner material.”	0.41	4.83	7 (15)
	Discomfort sleeping/at night	“Was uncomfortable during sleep.”	0.92	10.93	5 (11)
	EDA^b^ sensors were uncomfortable	“Circular sensors are too big and rub too much.”	1	11.87	3 (6)
	The monitor does not look good	“It could be a little more sleek and comfortable.”	0.59	6.95	1 (2)
**Positive/neutral statements about device**					
	Felt OK when wearing monitor	“No different than normal.”	0.47	5.62	35 (7)
	Could tolerate negatives	“It isn’t very comfortable but I managed.”	0.58	6.84	5 (11)
	Something positive about monitor's looks	“It looked cool.”	0.76	9.05	5 (11)
**Positive about paradigm/study**					
	Liked helping in a research study	“I felt I was helping.”	0.86	10.17	28 (60)
	Liked expressing distress	“I could press the button when I was in distress.”	0.94	11.15	7 (15)
	Helped become aware of distress	“I was able to be more alert and attentive to when I was having a hard time.”	1	11.87	3 (6)

^a^Kappa from initial coding round.

^b^EDA: electrodermal activity.

The most commonly endorsed codes were “feeling OK when wearing the monitor” (74.5% of the sample) and “liked helping in a research study” (59.6%). Interestingly, although the study only asked participants to monitor and express their distress to the extent needed to remember to press the button, some participants reported that they liked the monitoring because it allowed them to express distress (14.89%) or become more aware of distress (6.38%). On balance, nearly half of the participants reported some discomfort while wearing the device (46.8%), with the most common complaint that the device was too large (36.2%). As can be seen in Table 3, there were no significant differences between those who did or did not endorse any of the qualitative categories on hours worn per day or attrition status.

**Table 3:** Differences in daily hours worn and attrition status by qualitative category endorsement (codes for “device does not look good” and “helped become aware of distress” were not included in these analyses because of low frequency of endorsement).

Category	Hours worn				Attrition			
	Endorsed, mean (SD)	Did not endorse, mean (SD)	*t* test^a^	*P* value	Did not drop out, n (%)	Dropped out, n (%)	*χ*^2^ value *(df)*	*P* value
Discomfort/uncomfortable in general	14.05 (6.98)	15.96 (5.49)	0.88	.39	19 (45)	4 (57)	0.35 *(1)*	.55
Discomfort sleeping/at night	16.19 (5.25)	15.01 (6.32)	−0.42	.69	5 (12)	0 (0)	0.98 *(1)*	.32
EDA^b^ sensors were uncomfortable	11.28 (9.55)	15.5 (5.83)	0.75	.52	2 (5)	1 (14)	0.86 *(1)*	.35
The monitor was too big/clunky	16.37 (4.73)	14.41 (6.85)	−1.00	.32	16 (37)	2 (29)	0.21 *(1)*	.65
The material was uncomfortable	11.03 (8.13)	15.83 (5.64)	1.27	.26	5 (12)	2 (29)	1.21 *(1)*	.27
It should have a clock	15.99 (5.40)	14.85 (6.46)	−0.52	.61	10 (22)	2 (29)	0.12 *(1)*	.73
Felt OK when wearing monitor	15.70 (5.49)	13.75 (7.70)	−0.73	.48	33 (78)	4 (57)	1.30 *(1)*	.25
Could tolerate negatives	17.90 (1.83)	14.79 (6.43)	−2.11	.05	5 (13)	0 (0)	0.98 *(1)*	.32
Something positive about monitor’s looks	14.07 (4.40)	15.28 (6.38)	0.49	.64	4 (10)	1 (14)	0.12 *(1)*	.73
Liked expressing distress	12.13 (8.75)	15.64 (5.65)	0.87	.43	5 (13)	2 (29)	1.21 *(1)*	.27
Liked helping in a research study	16.32 (5.46)	13.14 (6.93)	−1.41	.17	25 (58)	5 (71)	0.48 *(1)*	.49

^a^All values have 1 degree of freedom *(df)*.

^b^EDA: electrodermal activity.

## Discussion

### Principal Findings

This study examined and found support for the feasibility and acceptability of using wearable behavioral and psychophysiological monitors to continuously collect objective data from adolescents with clinically severe psychiatric problems. There are several key findings from this study. First, participants were compliant with wearing the monitor, doing so on average more than 18 hours per day. Second, participants were compliant with instructions to use the event marker when distressed and were able to do so independently and without prompting. Third, participants found wearing the monitor to be acceptable and liked wearing them because it was part of a research study (eg, helping researchers understand psychiatric conditions that might someday be used to help others in a similar position). We discuss these main findings in greater detail below.

### Do Participants Wear the Monitor?

The first aim of the study was to assess how often participants wore the monitor. We found that participants wore the E4 at some point nearly every day and did so, on average, more than 18 hours a day. We also found that the variability in hours worn per day has more to do with daily-level factors than with person-level factors. These data reflect that it may not be that there are certain types of participants who wear wristband more than others, but rather certain days where any given participant is more likely to wear the wristband than other days. Accordingly, future research could focus on reasons for this day-to-day variability to optimize participant compliance (eg, by finding factors that can identify the types of days where participants are more or less likely to wear the wristband). Overall, these data suggest that it is possible to use wearable monitors to collect continuous, objective data from clinically severe adolescents as they navigate their daily lives, and as such, to collect exponentially larger and more ecologically valid data than what has been possible in laboratory studies where recordings might be for a few hours at most while performing benign experimental tasks. This opens up myriad possibilities for better understanding the phenomenology and prediction of a range of clinical outcomes such as depression, anxiety, psychotic experiences, and suicidal and violent thoughts/behaviors.

### Do Participants Correctly Use the Event Marker?

The second aim of the study was to assess whether participants interacted with the monitor. We asked participants to press the event marker on the monitor when they were feeling distressed and found that they were generally compliant in doing so. Participants rarely accidentally pressed the button (approximately 1 in 216 button presses was an accident) and rarely forgot to press the button (participants forgot to press the button approximately 1 out of every 59 times they should have). This is particularly impressive, given the circumstances under which we asked participants to press the button. Being on an inpatient unit can be unfamiliar and stressful and being highly distressed (which is when they were asked to press the button) is an affect state, which may be particularly difficult to self-monitor and respond to. Indeed, the basis for this larger study these data are drawn from is to attempt to identify distress using the E4’s sensors so that interventions could be developed that do not rely on patients needing to monitor for distress. Regarding the frequency of the button presses each day, we found that although there was day-to-day variability in the times each participant pressed the button each day, there was more variability from participant to participant, suggesting that some participants tended to use the event marker more than others. It is unclear whether this is best explained by between-participant variability in conscientiousness in pressing the button versus in the likelihood of actually experiencing distress. Future work examining the correspondence between button presses and actual physiological arousal (ie, as an objective indicator of distress) will help to address this question. We also found that as the number of days in the study increased, the number of times pressing the button each day slightly decreased. This could be a marker of an effective treatment (ie, participants were feeling less distressed) or a marker of study fatigue. Given how infrequently participants did not press the button when they should have, it is probably more likely that this is a marker of treatment efficacy rather than study fatigue.

### Do Participants Like Wearing the Monitor?

The third aim of the study was to assess both quantitively and qualitatively what participants liked and did not like about participating in a study using wearable physiological monitors. We found that participants tended to report that the monitor did not interfere much with their movement and that they *felt OK* when wearing the monitor. Many participants reported that one of the aspects they liked the most about the study was being in a study where they could contribute to knowledge acquisition and/or help others similar to them. On balance, nearly half of the sample noted that the monitor was generally uncomfortable, with more than one-third of participants noting the monitor was too bulky. It is also notable that more than 15% of participants said that they liked how the monitor helped them be more aware of their distress, although this was not an explicit goal of the study. This is interesting because it may suggest the viability of interventions using these monitors to help participants self-monitor and manage their own distress in a more explicit manner.

Taken together, these findings suggest that doing research with these wearable monitors is feasible, especially when participants are motivated to be in a study. These findings also suggest, however, that the feasibility of using wearable physiological monitors for research may not translate to applied settings where the incentive of being in a research study is not there to motivate compliance. In these cases, there would be fewer incentives to balance out the negatives about the size of the monitor. Thus, more comfortable monitors may be required to obtain similar compliance to what we found in this study. For example, the Empatica Embrace is a currently available consumer monitor that is approximately 30% smaller than the E4 (30×30×10 mm and 44×40×16 mm, respectively). Although not explicitly designed for research use, it can be used in some research applications (eg, studies concerned with movement and skin conductance, but not HR because it does not have a PPG sensor). Moreover, it is reasonable to expect that smaller research-grade monitors will be available from a variety of manufacturers in the near future.

### Limitations, Strengths, and Future Directions

These findings should be interpreted in the context of 3 key limitations. First, although we assessed the *amount* of data received, we did not assess the *quality* of data received. Not all data collected from any wearable monitor are usable (eg, because of motion artifacts). Evaluating the quality of the data obtained in such studies is an important step for future research, and this is something that we will be undertaking in our ongoing work in this area. Second, we used recording length as a proxy for time wearing the E4. If the E4 is turned on, it records even if it is not being worn. Thus, it is possible that the data reported here on time that the monitor was worn may be overestimated. There are 2 reasons why the probability of time worn overestimations is quite low. One is that participants were told to turn off the E4 whenever it was not being worn. Another is that data were collected on a psychiatric inpatient unit where unit staff observes patients at least once every 5 min. If unit staff saw an unworn E4 that was powered on, they would turn off the monitor. A third limitation, related to the previous point, is that these data were collected on an inpatient unit where participants were continually monitored by clinical staff and visited nearly daily by research staff, possibly making them more likely to wear the monitors. Thus, findings from this study may not generalize to other settings where there is less intensive adult supervision (eg, schools and home) or to other samples (eg, adults). Examining the generality of these results in other settings and samples is another important future step for research in this area. Finally, although our study provided a rich description of how often participants wore the monitor and interacted with it, the study was unable in some cases to provide explanations of why participants wore a device more on some days than others. Similar to many studies whose goal is description, future studies should explore possible explanations for the phenomena described in this study.

In conclusion, the clearest implication from this study is that it is feasible to conduct research where participants wear physiological monitors for an extended period (ie, days or weeks). This implication is in line with other studies of samples of adults with less severe psychopathology [21]. The study’s findings are important because studies that use wearable behavioral and psychophysiological monitors over an extended period have great promise to help researchers understand how constructs of interest to psychological scientists operate in everyday life. This is especially true for studies that combine these data streams with other streams of data such as medical records, passive mobile phone sensing, and ecological momentary assessment. Our ability to conduct studies similar to this is only just beginning, and it is undoubtable that wearable technology will become even more advanced in the coming years, making studies even more feasible in the future.

## Abbreviations

EDA: electrodermal activity
HR: heart rate
ICC: intraclass correlation
PPG: photoplethysmograph
